# BRINGING GERIATRIC CARE INTO THE HIV PRIMARY CARE CLINIC: A COMBINED HIV/GERIATRIC MULTIDISCIPLINARY CLINIC MODEL

**DOI:** 10.1093/geroni/igad104.0382

**Published:** 2023-12-21

**Authors:** Angela Condo, Abigail Baim-Lance, Nathan Golstein

**Affiliations:** Icahn School of Medicine at Mount Sinai, New York City, New York, United States; Veterans Health Administration, Bronx, New York, United States; Department of Geriatrics and Palliative Care, Mount Sinai, New York, New York, United States

## Abstract

Persons living with HIV (PLWH) experience accelerated and accentuated aging, developing co-morbidities at earlier ages and with higher frequencies than those without HIV. Older persons with HIV (OPWH) also experience geriatric syndromes. In 2020 we developed a combined HIV/geriatrics multidisciplinary clinic at Mount Sinai Hospital in New York where a geriatrician and multidisciplinary team including a nurse, pharmacist, and social worker are embedded in an HIV primary care clinic. Patients are referred for comprehensive geriatric assessments. We conducted a retrospective chart review after the first 15 months of the program to describe this population of OPWH living in NYC who are referred to a geriatrician. From 9/2020-11/2021, there were 183 appointments scheduled of which 112 (61%) were new patient visits. Sixty-two percent of patients came to their appointment. The average age of patients was 67 years. Fifty-seven percent identified as male. The majority of referrals (50%) were for cognitive assessment followed by mobility assessment (25%), polypharmacy (14%), multimorbidity (8%), advance care planning (1%), and other (2%). Based on this data, we identified a need to improve the show rate to our clinic and implement cognitive screening in OPWH. We have incorporated a community health worker into our interdisciplinary team to assist in this goal and continue to educate patients and providers on geriatric syndromes. A geriatric approach to care is crucial to incorporate into existing HIV clinical practices and we hope to optimize this integration based on the needs of patients and providers.

